# Impact of Functional Frailty Measured by Short Physical Performance Battery Test on the Effectiveness of SGLT2 Inhibitors in Elderly Patients with Acute Heart Failure:PROFUND-IC Registry

**DOI:** 10.3390/jcm15207769

**Published:** 2026-10-09

**Authors:** Alicia Guzmán-Carreras, Purificación Romero-Jiménez, Sahar Okab, Rafael Sánchez-del Hoyo, Aladin Abdelhady Kishta-Kishta, Juan Igor Molina-Puente, Fernando Aguilar-Rodríguez, Noel Lorenzo-Villalba, Emmanuel Andrès, María del Rosario Iguarán-Bermúdez, Manuel Méndez-Bailón

**Affiliations:** 1 Department of Internal Medicine, Hospital Clínico San Carlos, 28040 Madrid, Spain; 2 Faculty of Medicine, Universidad Complutense de Madrid, 28040 Madrid, Spain; 3 Health Research Institute, Hospital Clínico San Carlos (IdISSC), 28040 Madrid, Spain; 4 Research Methodological Support Unit and Preventive Department, Hospital Clínico San Carlos, 28040 Madrid, Spain; 5 Department of Internal Medicine, Complejo Asistencial de Ávila, 05071 Ávila, Spain; 6 Department of Internal Medicine, Hospital 12 de Octubre, 28041 Madrid, Spain; 7 Department of Internal Medicine, Hôpitaux Universitaires de Strasbourg, 67200 Strasbourg, France

**Keywords:** acute heart failure, sodium-glucose cotransporter type 2 inhibitors, Short Physical Performance Battery (SPPB) test, frailty

## Abstract

**Background/Objectives:** Acute heart failure (AHF) is the leading cause of hospitalization among patients over 65 years and is frequently associated with frailty. Sodium-glucose cotransporter type 2 inhibitors (SGLT2i) currently represent the only drug class recommended across the entire ejection fraction spectrum, although evidence regarding their effectiveness according to frailty status remains limited. **Aims:** To determine whether functional frailty, measured by the Short Physical Performance Battery (SPPB) test, influences the effectiveness of SGLT2i in reducing hospital readmission and mortality in elderly patients with AHF. **Methods:** Observational study of 794 polypathological patients admitted to Internal Medicine with AHF. An interaction analysis assessed whether SPPB, as a continuous variable, modified the association between SGLT2i and the composite outcome (hospital readmission or death), using a Cox model. The sample was further divided by SPPB test score (below 4 (*N* = 477) or ≥4 (*N* = 317)) for exploratory analyses. Descriptive and comparative analyses were performed according to SGLT2i administration, using the chi-squared test for qualitative variables and Student’s *t*-test for quantitative ones (Fisher’s and Mann–Whitney U test as appropriate). Survival analysis was done using Kaplan–Meier curves and Cox regression, with multivariable adjustment for confounders. **Results**: No significant interaction was found between SPPB score and SGLT2i (*p* = 0.506). When SPPB was dichotomised, SGLT2i treatment was associated with significantly lower all-cause mortality (3.7% vs. 12.0%; *p* = 0.018) and readmission (30.3% vs. 45.2%; *p* = 0.021) in SPPB ≥ 4, with mortality and readmission also numerically lower, without reaching statistical significance, in SPPB < 4 (mortality 12.9% vs. 16.9%, *p* = 0.410; readmission 34.5% vs. 42.0%, *p* = 0.220). After adjustment, SGLT2i treatment in the SPPB ≥ 4 group remained associated with a numerically lower hazard for the composite outcome (HR 0.652; 95% CI 0.418–1.018; *p* = 0.060). **Conclusions:** No evidence was found that functional frailty modifies the effectiveness of SGLT2i in elderly polypathological patients with AHF. An exploratory analysis suggested a possible gradient of greater association with SPPB ≥ 4, although this should be interpreted as hypothesis-generating.

## 1. Introduction

Heart failure (HF) is a clinical syndrome characterized by symptoms and signs, as well as abnormalities in imaging and laboratory tests resulting from pulmonary or systemic congestion and/or impaired cardiac output, that are caused by a structural or functional heart condition [1]. It is a highly prevalent disease, affecting more than 64 million people worldwide, and constitutes the leading cause of hospitalization among patients over 65 years [2]. In this age group, one-year mortality following hospital discharge reaches 35% [3]. Furthermore, HF is associated with a substantial impairment in quality of life and 30-day readmission rates around 20–25% [4].

Heart failure is frequently associated with frailty, defined as a decline in functional reserve leading to an inadequate response to stressors [5]. In turn, frailty worsens the prognosis of heart failure as it is linked to an increased risk of both hospitalisation and mortality [6]. In this context, the Short Physical Performance Battery test (SPPB) is a validated tool for assessing physical frailty or functional status, evaluating three domains: standing balance, walking speed, and chair stand test, using a score ranging from 0 to 12 [7]. Several studies have demonstrated its prognostic value in heart failure, with lower SPPB scores associated with a higher risk of mortality and readmission [8,9,10].

Regarding the treatment of heart failure, sodium-glucose cotransporter type 2 inhibitors (SGLT2i) currently represent the only class of drug recommended across the entire ejection fraction spectrum [11,12,13]. Although their efficacy and tolerability are comparable regardless of age, therapeutic inertia remains a barrier to their implementation, partly due to the limited evidence available in frail patients [3,14]. This is reflected in a recent nationwide Danish study, in which frailty was associated with a significantly lower likelihood of initiating SGLT2i therapy [14]. Nevertheless, the available evidence has consistently shown that the effectiveness of SGLT2i is maintained irrespective of frailty severity assessed by the Rockwood Frailty Index [15,16,17]. However, no comparable studies have evaluated this question using alternative functional assessment tools.

Therefore, the primary aim of the present study was to determine whether functional frailty measured by the SPPB test influences the effectiveness of SGLT2i in reducing hospital readmission and mortality in elderly patients with acute heart failure.

## 2. Materials and Methods

### 2.1. Study Design

This is an observational study of 794 polypathological patients admitted to the Department of Internal Medicine with a primary diagnosis of acute heart failure. Data were obtained from the prospective, multicentre PROFUND-IC registry, created through the collaboration between the Heart Failure and Atrial Fibrillation Working Group and the Polypathological Patient and Advanced Age Working Group of the Spanish Society of Internal Medicine.

### 2.2. Study Population

Patients enrolled in the registry between October 2020 and 31 August 2024 who met the inclusion and exclusion criteria were selected.

### 2.3. Inclusion Criteria

- Patients aged 18 years or over admitted to the Department of Internal Medicine with a primary diagnosis of acute heart failure. The term “acute heart failure” encompasses both new-onset heart failure (de novo HF) and acutely decompensated chronic heart failure.
- To have an NT-proBNP > 1500 pg/mL on admission.
- Polypathological patient status, defined as the presence of two or more diseases included in the Ollero criteria [18].
- Available information regarding sodium-glucose cotransporter type II inhibitors (SGLT2i) prescription. Patients were considered treated with SGLT2i if they were receiving this treatment prior to admission and maintained it throughout hospitalisation and at discharge, or if they initiated SGLT2i within the first 72 h of the acute heart failure episode and maintained it at discharge. Patients who initiated SGLT2i within this 72-h window but died during hospitalisation, before reaching discharge, were likewise classified as treated based on having received the drug during admission, thereby avoiding immortal-time bias. Information on potential treatment discontinuation after discharge was not available, as follow-up visits recorded only readmissions and mortality events.
- The SPPB test must have been performed during their hospital stay (within the first 72 h of admission once the patient had achieved sufficient haemodynamic stability to safely perform the test).
- Completion of the 12-month clinical follow-up.

The only exclusion criterion was refusal to participate in the study or to sign the informed consent form by the patient or their legal representative.

Although the general enrolment criterion of the PROFUND-IC registry allows the inclusion of adult patients aged ≥ 18 years, in clinical practice, patients admitted with acute heart failure and significant polypathology to Internal Medicine departments are predominantly older adults. Accordingly, the mean age of the present cohort was 83.9 years (SD 9.0), which supports the use of the term ‘elderly’ throughout the manuscript.

Of the 870 patients meeting the general enrolment criteria of the PROFUND-IC registry who consented to participate, 76 (8.7%) were excluded due to missing SPPB assessment, resulting in a final sample of 794 patients. As completion of 12-month follow-up was also required for inclusion, the available dataset did not allow losses to follow-up to be quantified as a category separate from missing SPPB assessment.

### 2.4. Study Variables

SGLT2i administration was analysed as the primary exposure.

Given the relatively low number of deaths anticipated in this cohort, which would limit statistical power if mortality were analysed in isolation, the primary outcome was defined as a composite time-to-first-event outcome (hospital readmission or death, whichever occurred first), used for the Cox regression survival analysis addressing the study’s principal research question. Each patient contributed a single event to this analysis, corresponding to the time to their first qualifying event during the 12-month follow-up period. If readmission occurred before death, the patient was counted as reaching the composite outcome via readmission, regardless of whether death occurred subsequently within the follow-up period. No patient was counted more than once.

The sample was then divided into two groups according to SPPB score (below 4 (*N* = 477) or greater than or equal to 4 (*N* = 317)). Exploratory ROC curve analyses were performed separately for the two components of the composite outcome, after examining the available data. For hospital readmission, the area under the curve (AUC) was 0.497 (95% CI 0.457–0.537; *p* = 0.876), indicating no discriminative capacity of SPPB for this outcome. For all-cause mortality, the AUC was 0.610 (95% CI 0.556–0.665; *p* < 0.001), suggesting an optimal cut-off of 5.5 points, although reflecting poor discriminative performance. Consequently, the sample was dichotomised using the threshold for the category of ‘severe functional limitation or severe physical frailty’ (SPPB < 4), as originally described by Guralnik et al. [19] and subsequently validated by the meta-analysis of Pavasini et al. [7], in which this category showed the strongest association with all-cause mortality (OR 3.25 compared with the reference category 10–12). This approach also allowed for a more balanced sample size distribution between groups.

Patients’ epidemiological characteristics were also recorded (sex, age (years), and body mass index (BMI, kg/m^2^)), along with their comorbidities (diabetes mellitus, dyslipidaemia, hypertension, atrial fibrillation, ischaemic heart disease, chronic respiratory disease, chronic kidney disease, cerebrovascular disease, neurological disease with permanent motor impairment, chronic liver disease, peripheral artery disease, solid or haematological malignancy, osteoarticular disease, and cognitive impairment).

With respect to heart failure, New York Heart Association (NYHA) functional class and left ventricular ejection fraction (LVEF, %) were included.

Given the age and comorbidity burden of the patients, the Barthel Index, the Mini Nutritional Assessment Short Form (MNA-SF), the Rockwood frailty index > 4, median SPPB score, and handgrip strength (kg) are also described.

Analytical variables were also obtained (creatinine (mg/dL), NT-proBNP (pg/mL), CA-125 (IU/mL), cholesterol (mg/dL), and albumin (g/dL)), as well as established treatment (maximum furosemide dose received (mg/day), angiotensin-converting enzyme inhibitors (ACEIs), angiotensin II receptor blockers (ARBs), angiotensin receptor-neprilysin inhibitors (ARNIs), beta-blockers, sodium-glucose cotransporter-2 inhibitors (SGLT2i), mineralocorticoid receptor antagonists (MRAs), and protein supplements).

All functional and analytical variables, including the SPPB test, were collected within the first 72 h of admission. The SPPB was assessed once the patient had achieved sufficient haemodynamic stability to safely perform the test. All participating investigators are trained in the SPPB and other comprehensive geriatric assessment tools. Laboratory variables were measured according to routine clinical practice at each participating centre, and handgrip strength was measured with the hand dynamometer available at each centre. NYHA functional class refers to the patient’s baseline status prior to the acute decompensation episode, reflecting chronic rather than acute severity. LVEF was obtained from the echocardiogram performed during the index admission when available, or from the most recent prior echocardiogram if none was performed during this admission. Maximum furosemide dose refers to the highest dose received at any point during the entire hospital stay.

Finally, the number of deaths and readmissions over the following 12 months were analysed as prognostic variables in each group.

### 2.5. Statistical Analysis

The primary outcome was the composite of all-cause hospital readmission or all-cause mortality, whichever occurred first, over 12 months of follow-up. The primary analysis assessed whether the association between SGLT2i use and the primary outcome varied according to SPPB score, by means of a Cox model incorporating an interaction term between SGLT2i use and SPPB. SPPB test was modelled as a continuous variable using restricted cubic splines. Treatment hazard ratios were calculated across the observed range of SPPB scores and plotted graphically. The interaction *p*-value was obtained by comparison of nested models with and without the interaction term.

Given the absence of a statistically significant interaction, secondary, exploratory analyses were performed to further characterise the pattern of the association across the frailty spectrum. The sample was then divided into two groups according to SPPB test score: below 4 (*N* = 477) and greater than or equal to 4 (*N* = 317). A descriptive analysis was performed within each group. Quantitative variables are presented as mean and standard deviation (or median and interquartile range where normality was not met). Qualitative measures are likewise described as absolute number and percentage.

A comparative analysis was carried out within each group according to SGLT2i use, using the chi-squared test for qualitative variables and Student’s *t*-test for quantitative variables (with Fisher’s exact test and the Mann–Whitney U test applied when appropriate).

Finally, survival analysis (all-cause mortality and all-cause hospital readmission separately) was performed using Kaplan–Meier curves, with groups compared according to SGLT2 inhibitor use by means of the log-rank test. For the Kaplan–Meier analysis of hospital readmission, patients who died without a prior readmission were censored at the time of death; this standard approach does not formally account for the competing risk of death, although the majority of patients who died had already experienced a readmission beforehand, limiting the practical impact of this competing risk in our cohort.

A crude (unadjusted) Cox regression was first performed for the primary (composite) outcome. The analysis was then completed using multivariable Cox regression. Variables were selected based on statistically significant differences (*p* < 0.10) between the treated and untreated groups, combined with established clinical and prognostic relevance in heart failure and concomitant background therapy, in order to minimise the possibility that the observed association reflected differences in overall treatment intensity rather than SGLT2i use itself. This resulted in the inclusion of sex, age, body mass index, diabetes mellitus, atrial fibrillation, cognitive impairment, neurological disease with permanent motor impairment, solid or haematological malignancy, NT-proBNP levels, LVEF, use of ARNIs, beta-blockers and mineralocorticoid receptor antagonists, and maximum daily furosemide dose.

Given the low and unevenly distributed prescription rate of ARNI between SGLT2i-treated and untreated patients (the largest imbalance observed among all covariates considered (21.1% vs. 5.3% in the SPPB ≥ 4 subgroup; *p* < 0.001)) and the relatively limited number of events available for multivariable adjustment, an additional exploratory sensitivity analysis excluding ARNI use from the fully adjusted model was performed post hoc, motivated by this observed covariate imbalance rather than by the result of the sensitivity analysis itself. The potential confounding effect of each covariate was also assessed individually by incorporating it separately into a Cox model containing SGLT2i treatment alone.

As a sensitivity analysis, an overlap-weighting propensity-score approach was additionally performed using the same covariates, in order to further address potential confounding by indication while preserving sample size. This analysis was conducted both including and excluding sacubitril/valsartan use, given its potential collinearity with SGLT2i prescription.

Missing data proportions for all study variables are reported in Supplementary Table S5. Missing data were below 5% for all covariates included in the multivariable models.

Statistical analyses were performed using IBM SPSS Statistics version 26.0 (IBM Corp., Armonk, NY, USA) and R version 4.6.1 (R Foundation for Statistical Computing, Vienna, Austria).

### 2.6. Ethical Considerations

The PROFUND-IC registry was approved by the Ethics Committee of Hospital Fundación Alcorcón. The study was conducted in accordance with the principles of the Declaration of Helsinki. All patients, or their legal representative, have signed the informed consent form after properly explained to them. The authors declare no conflicts of interest.

## 3. Results

**Figure 1:**
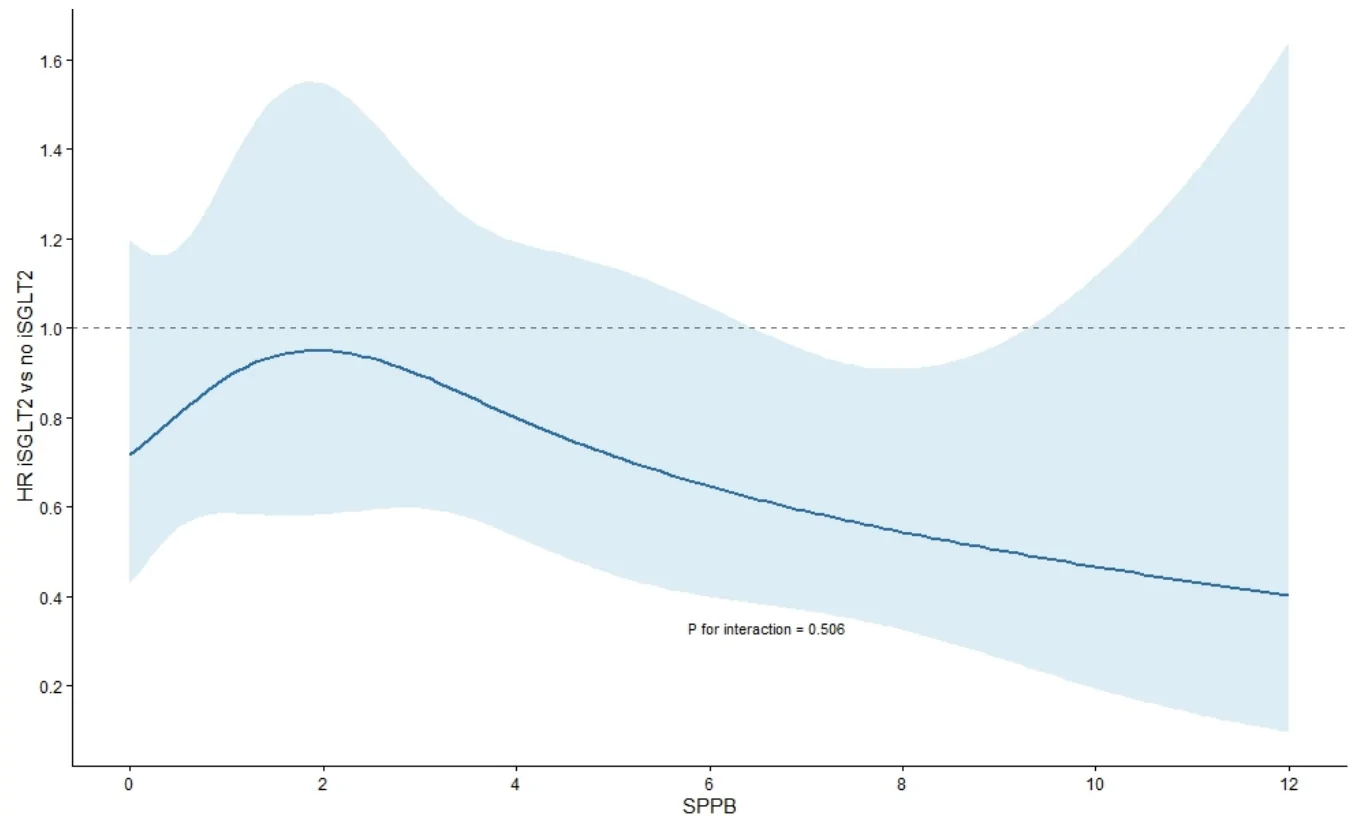
Association between SGLT2i use and the primary (composite) outcome in elderly patients with acute heart failure according to SPPB test. The solid blue line represents the estimated hazard ratio (HR) of SGLT2i use versus no SGLT2i use, with SPPB modelled as a continuous variable. The shaded blue area represents the 95% confidence interval. The dashed horizontal line indicates HR = 1.0 (no association). *p* for interaction = 0.506.

The interaction analysis using the SPPB as a continuous variable showed no evidence of modification of the association (*p* for interaction = 0.506), suggesting that the benefit of SGLT2i does not differ according to the degree of physical performance measured by SPPB test. Nevertheless, the estimated association appeared to become progressively greater with increasing SPPB scores (Figure 1).

Given the absence of a significant interaction, subgroup analyses stratified by SPPB category (≥4 vs. <4) are presented below to characterise the pattern of effect across the frailty spectrum.

### 3.1. Patients with SPPB Test < 4

A total of 477 patients with an SPPB score < 4 were included, of whom 139 (29.1%) were receiving SGLT2i. Overall, 302 (63.3%) were women, with a mean age of 85.6 years and a median Barthel Index of 60. The most common comorbidities were hypertension (434, 91%), atrial fibrillation (351, 73.6%) and dyslipidaemia (303, 63.5%). Most patients were classified as NYHA functional class II (239, 50.1%) with a mean LVEF of 51.8%. During follow-up, 75 patients died (15.7%) and 190 (39.8%) required readmission (Table 1).

**Figure 2:**
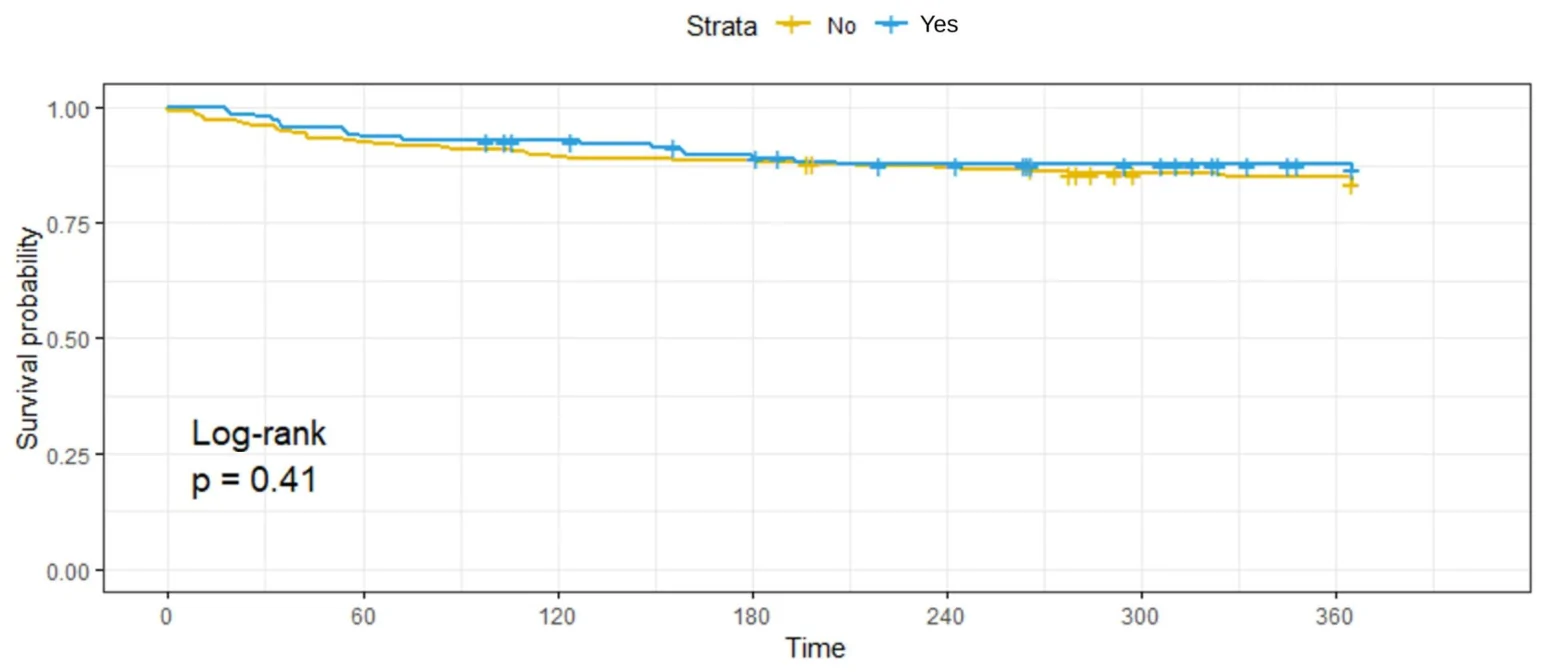
Overall survival of patients with SPPB < 4 according to treatment with SGLT2i.

**Figure 3:**
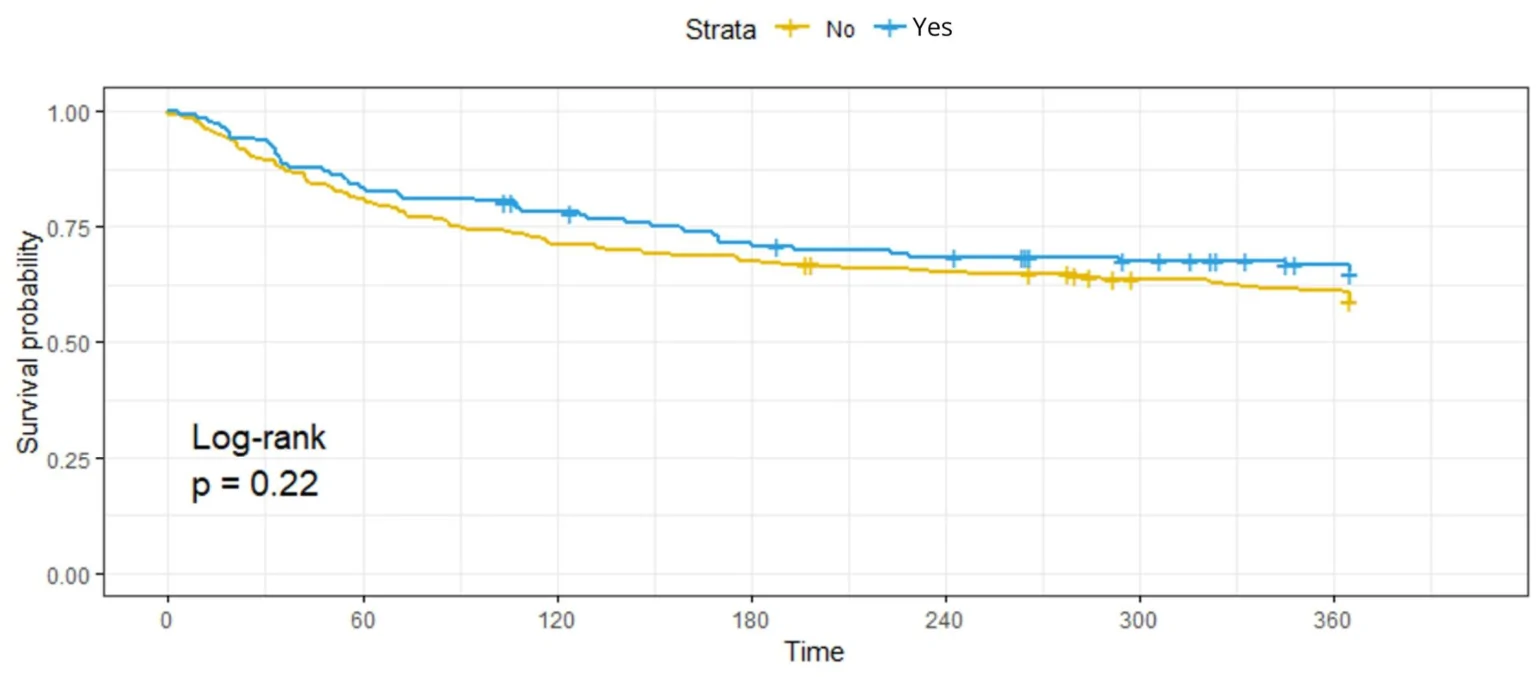
Readmission rate in patients with SPPB < 4 according to SGLT2i treatment.

Regarding differential characteristics according to SGLT2i treatment, no significant differences were observed between the groups in terms of sex or age. Patients receiving SGLT2i more frequently had diabetes mellitus (57.6% vs. 42.3%, *p* = 0.002) and showed less cognitive impairment (7.2% vs. 15.7%, *p* = 0.013). No significant differences were observed between the groups in NYHA class, LVEF, or other functional assessment scales. There was a non-significant trend towards a higher median NT-proBNP level in the treated group (6854 vs. 5624 pg/mL, *p* = 0.073), whereas patients receiving SGLT2i had a significantly lower mean cholesterol level (132.6 vs. 141.6 mg/dL, *p* = 0.014). Furthermore, the treated group more frequently received concomitant ARNI (12.2% vs. 4.4%, *p* = 0.002), beta-blockers (66.2% vs. 55.9%, *p* = 0.038), MRAs (43.9% vs. 27.2%, *p* < 0.001) and higher doses of furosemide (100 vs. 80 mg/day, *p* = 0.010). All-cause mortality was numerically lower among patients receiving SGLT2i (12.9% vs. 16.9%; *p* = 0.410) (Figure 2), without reaching statistical significance, as was hospital readmission (34.5% vs. 42.0%; *p* = 0.220) (Figure 3) (Table 1). In absolute terms, this corresponded to a risk difference of −4.0 percentage points for mortality and −7.5 percentage points for readmission. The crude Cox regression for the composite outcome (readmission or death) in this subgroup showed a hazard ratio of 0.813 (95% CI 0.584–1.134; *p* = 0.223).

### 3.2. Patients with SPPB Test ≥ 4

A total of 317 patients with a SPPB test score of ≥4 were included. Of these, 109 (34.4%) were receiving SGLT2i. 163 (51.4%) were men, with a mean age of 81.4 years and a median Barthel Index of 95. The most frequent comorbidities were hypertension (284, 89.6%), atrial fibrillation (207, 65.3%) and dyslipidaemia (196, 61.8%). Most patients were classified as NYHA functional class II (190, 59.9%) with a mean LVEF of 51%. During follow-up, 29 patients (9.1%) died and 127 (40.1%) were readmitted to hospital (Table 2).

**Figure 4:**
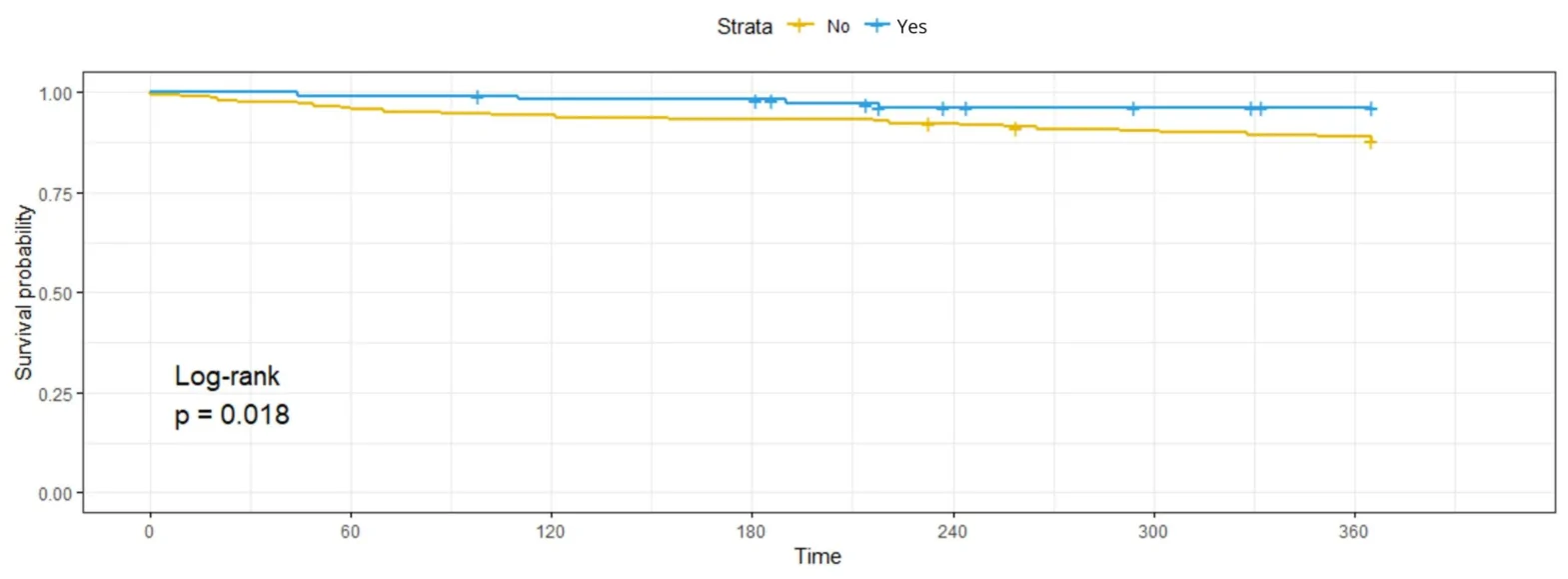
Overall survival of patients with SPPB ≥ 4 by SGLT2i treatment.

**Figure 5:**
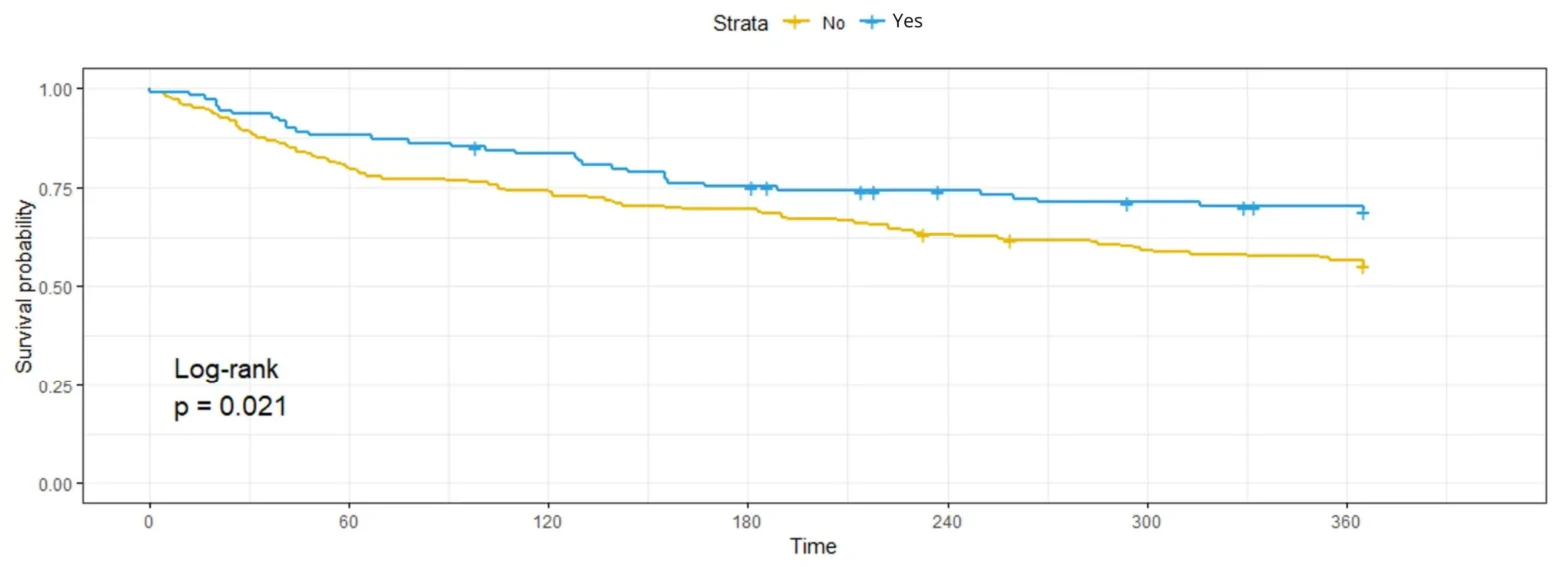
Readmission rate of patients with SPPB ≥ 4 by SGLT2i treatment.

Regarding differences according to treatment with SGLT2i, patients receiving SGLT2i were more frequently male (62.4% vs. 45.7%; *p* = 0.005), with lower mean age (80.1 vs. 82.1, *p* = 0.078) and higher body mass index (29.4 vs. 27.9, *p* = 0.032). Those who received SGLT2i were more likely to have diabetes mellitus (59.6% vs. 34.6%, *p* < 0.001) and atrial fibrillation (73.4% vs. 61.1%, *p* = 0.028) whereas active malignancy was less common (1.8% vs. 10.1%, *p* = 0.007). No significant differences were observed in NYHA functional class. However, LVEF was lower in the treated group (47.8% vs. 52.8%, *p* = 0.001). Patients receiving SGLT2i also had a significantly higher median NT-proBNP level (5849 vs. 4633 pg/mL, *p* = 0.005). Concomitant treatment with ARNI (21.1% vs. 5.3%; *p* < 0.001), beta-blockers (78.0% vs. 59.1%; *p* = 0.001), MRA (45.0% vs. 31.7%; *p* = 0.015), and higher maximum daily doses of furosemide (80 vs. 60 mg/day; *p* < 0.001) was significantly more frequent among the treated group. Patients with SGLT2i had significantly lower all-cause mortality (3.7% vs. 12.0%; *p* = 0.018) (Figure 4) and hospital readmission rates (30.3% vs. 45.2%; *p* = 0.021) (Figure 5) during one year follow-up (Table 2). In absolute terms, this corresponded to a risk difference of −8.3 percentage points for mortality and −14.9 percentage points for readmission.

In the unadjusted Cox regression survival analysis, patients with acute heart failure and SPPB ≥ 4 treated with SGLT2i had a 37% lower risk of experiencing the composite outcome (hospital readmission or death) during the one-year follow-up compared with those who did not receive the drug (HR 0.630; 95% CI 0.423–0.938; *p* = 0.023) (Table 3).

Then, a multivariate analysis was carried out, including those variables with significant differences (*p* < 0.10) between the treated and untreated groups (sex, age, body mass index, diabetes mellitus, atrial fibrillation, cognitive impairment, neurological disease with permanent motor impairment, solid or haematological malignancy, NT-proBNP levels, LVEF, use of ARNI, beta-blockers and MRA, as well as maximum daily doses of furosemide). After adjustment, treatment with SGLT2i was associated with a 34.8% reduction in the risk of the event, almost reaching statistical significance (95% CI: 0.418–1.018) (*p* = 0.060) (Table S1 and Table 3).

After excluding ARNI treatment from the adjustment model, SGLT2i therapy was associated with a 37.6% reduction in the risk of the composite outcome during follow-up (HR 0.624; 95% CI 0.401–0.972; *p* = 0.037) (Table S2 and Table 3).

Overall, among elderly polypathological patients with acute heart failure and SPPB ≥ 4, the crude analysis showed a statistically significant 37% reduction in the risk of the composite outcome associated with SGLT2i treatment. This association remained in the same direction, with a numerically similar hazard ratio, in the fully adjusted model, although statistical significance was not reached (HR 0.652; 95% CI 0.418–1.018; *p* = 0.060). In an exploratory sensitivity analysis excluding ARNI the association reached statistical significance (HR 0.624; 95% CI 0.401–0.972; *p* = 0.037).

An overlap-weighting sensitivity analysis (Supplementary Tables S3 and S4) yielded results consistent with the primary multivariable analysis. SGLT2i treatment remained associated with a numerically lower hazard for the primary outcome (HR 0.66; 95% CI 0.43–1.03; *p* = 0.066) (Table 4), which reached statistical significance in the exploratory analysis when sacubitril/valsartan was excluded from the model (HR 0.64; 95% CI 0.41–0.99; *p* = 0.044).

## 4. Discussion

The main finding of this study is that treatment with SGLT2i in elderly polypathological patients with acute heart failure was associated with a favourable prognostic pattern, with a numerical reduction in readmission and mortality at twelve months. No significant interaction was found between SPPB score and SGLT2i use (*p* = 0.506), indicating that functional frailty did not significantly modify this association. When SPPB was analysed as a qualitative variable in an exploratory analysis, a possible gradient towards a stronger association was observed in patients with SPPB ≥ 4.

It is noteworthy that the study population was of very advanced age, with a mean age exceeding 80 years. Although patients in the original clinical trials were considerably younger [20,21,22,23], previous studies have demonstrated the effectiveness of SGLT2i irrespective of age [24,25,26]. In a subanalysis of the EMPEROR-Preserved trial, advanced age (>80 years) was not associated with reduced efficacy or poorer tolerability of empagliflozin compared with younger age groups [24]. Likewise, in the ROSES-HF study, initiation of SGLT2i following admission for acute heart failure in patients over 75 years of age was associated with lower subsequent readmission [25]. However, current evidence suggests that therapeutic decision-making should be guided not solely by chronological age but rather by patient complexity, including multimorbidity and frailty. In the AGING-HF study, which included patients admitted for acute heart failure with a mean age of 90 years and a mean Charlson Index of 8.2, SGLT2i use was associated with lower all-cause mortality and readmission at one year [26].

Moreover, as noted in the introduction, post hoc analyses of the DAPA-HF [15] and DELIVER trials [16] found no evidence that frailty, measured by the Rockwood frailty index, modified the effectiveness of these drugs. In the present study, when interpreting the SPPB test score as a continuous variable, no significant interaction was observed between functional frailty measured using this scale and SGLT2i, in line with previous studies [15,16]. However, when the SPPB was analysed as a qualitative variable using a cut-off of 4, statistical significance was reached only in the group with SPPB ≥ 4, while the group with SPPB < 4 (classified as severe frailty according to Guralnik et al. [19]) showed numerically lower mortality and readmission rates among treated patients. The absence of statistical significance in this severely frail subgroup should not be interpreted as evidence of either treatment benefit or treatment inefficacy. It may instead reflect limited statistical power, wide confidence intervals, and genuine heterogeneity of treatment effect across frailty categories in this relatively small subgroup. Furthermore, these patients may require earlier intervention and a more comprehensive multidisciplinary approach addressing nutritional, functional, and social factors to achieve a clinically meaningful benefit. Overall, although the formal interaction analysis did not support effect modification by SPPB score, the magnitude of the estimated association appeared to increase progressively with higher SPPB scores. This may explain why statistical significance was achieved only in patients with SPPB ≥ 4 after dichotomising the scale, not because SGLT2i become ineffective in severe frailty, but because the magnitude of benefit appears to be attenuated in these patients. This interpretation is consistent with previous evidence, as the beneficial effects of empagliflozin were also attenuated among patients with severe frailty in the post hoc analysis of the EMPEROR-Preserved trial [17]. Notably, even in the SPPB ≥ 4 subgroup, statistical significance was not retained after full multivariable adjustment (HR 0.652; 95% CI 0.418–1.018; *p* = 0.060), indicating that a degree of uncertainty affects this subgroup’s association as well. Overall, these findings should be regarded as hypothesis-generating.

We cannot exclude a potential synergistic interaction between SGLT2i and ARNI, which remains hypothesis-generating and warrants confirmation in larger prospective cohorts. However, we consider it more likely that the attenuated significance observed in the fully adjusted model reflects a combination of factors like reduced statistical precision after adjustment, residual confounding not fully captured by the covariates included, and the low events-per-variable ratio in this model. ARNI use may reflect more intensive guideline-directed medical therapy and could act both as a marker of overall treatment intensity and as a confounding factor, given its strong association with SGLT2i prescription in this cohort, reinforcing the need for cautious interpretation of these results. Excluding ARNI from the adjusted model could plausibly bias the estimated association in either direction, removing genuine confounding if ARNI independently influences outcomes, or inflating the apparent SGLT2i association if ARNI instead reflects unmeasured favourable prognostic factors correlated with more comprehensive therapy. Notably, ARNI itself has been associated with reverse cardiac remodelling and improved functional capacity in HFrEF, including in patients with chronic cardiac resynchronisation therapy, in whom late ARNI initiation was associated with significant improvements in quality of life, exercise capacity, and left ventricular ejection fraction [27].

Frailty and HF share a common pathophysiological substrate, including sarcopenia, inflammation and neurohormonal activation, which together contribute to reduced physiological reserve and accelerated functional decline [28]. Beyond their cardiovascular and renal effects, SGLT2 inhibitors have also been shown to reduce systemic inflammation and oxidative stress [29]. These findings are further consistent with a recent narrative review on frailty in heart failure, which highlighted that SGLT2 inhibitors have shown promise in frail HF patients, improving cardiovascular and renal outcomes and reducing hospitalisation rates [28]. The same review emphasised that effective frailty management in heart failure requires a multidimensional approach integrating physical, nutritional and psychosocial interventions, underscoring the relevance of the SPPB’s exclusively physical focus [28]. Particularly, malnutrition is common and prognostically significant in elderly patients with HF, independently associated with higher mortality [30]. Furthermore, malnutrition alone has been shown to reduce life expectancy irrespective of BMI category, reflecting an obesity paradox in which BMI alone may inadequately capture nutritional risk [31]. Although this study did not systematically assess nutritional status using validated indices such as the PNI or GNRI, exploratory analyses stratified by MNA-SF category did not identify statistically significant differences in the SGLT2i-associated pattern according to nutritional status. However, given that MNA-SF data were missing in a substantial proportion of the cohort (44.6%) and that these stratified analyses were limited by small event numbers, these findings do not allow us to exclude a possible modification of the association by nutritional status.

This study has several limitations:

- Study design. This is an observational, non-randomised study. Therefore, residual confounding may exist (including confounding by indication) despite the multivariable analysis performed. As this is not a randomised clinical trial, the study cannot establish treatment efficacy or exclude a clinically relevant effect modification.
- Statistical power. The relatively small sample size and low number of events, particularly in the severely frail subgroup, limit statistical power. Accordingly, these results should be understood as showing no evidence that severe functional frailty significantly modifies the association between SGLT2i treatment and clinical outcomes, rather than as proof that no such modification exists.
- SPPB cut-off and construct. Although the cut-off of 4 is supported by the literature for identifying patients with severe frailty and provides balanced, comparable subgroup sizes, it was not the optimal threshold according to the ROC curve derived from our cohort. Furthermore, the SPPB assesses only the physical domain of frailty and does not capture cognitive, psychological, social, or nutritional dimensions also recognised as relevant to frailty status and prognosis in heart failure.
- Exposure definition. Information on the specific SGLT2 inhibitor used and treatment dose was unavailable and may have influenced the observed outcomes. Information on treatment discontinuation after discharge was also unavailable. Patients classified as treated were assumed to remain exposed throughout follow-up, which may have led to some exposure misclassification, plausibly biasing the estimated association towards the null.
- Model specification. The ratio of events to covariates in the fully adjusted Cox model (approximately 7.9 events per variable) was below the conventionally recommended threshold of 10, raising concerns about model overfitting. This concern was particularly relevant for ARNI, given its low and unevenly distributed prescription rate between groups, motivating an exploratory sensitivity analysis excluding this variable, performed post hoc. Nonetheless, the crude and adjusted hazard ratios, the individual covariate-confounding assessment, and the propensity-score overlap-weighting sensitivity analysis showed similar magnitude and direction, although statistical uncertainty persists across these analyses. Additionally, because SGLT2i could be initiated within the same 72-h window used for functional and analytical assessments, and because LVEF was in some cases derived from a prior echocardiogram rather than one performed during the index admission, we cannot fully exclude the possibility that some baseline assessments were influenced by treatment initiation or reflected a clinical status prior to the current episode. Similarly, maximum furosemide dose refers to the highest dose received at any point during the entire hospital stay and may, in some patients, have been determined after SGLT2i initiation, representing a potential post-exposure variable. Its inclusion as a covariate may therefore have led to some degree of overadjustment, without fully eliminating confounding by indication. Additionally, although completion of 12-month follow-up was an inclusion criterion, we were unable to separately quantify the number of patients lost to follow-up independently of missing SPPB assessment using the available dataset. This precludes a fully detailed patient flow diagram and should be considered when interpreting the completeness of follow-up in this cohort.
- Competing risk. The Kaplan–Meier analysis of readmission did not formally account for death as a competing risk, following standard practice in the heart failure literature. However, as most patients who died had already been readmitted beforehand, the practical impact of this limitation on our readmission estimates is likely to be small.
- Registry-related. The PROFUND-IC registry began in 2020, which would explain the low utilisation rate of this drug class and the consequent limitation in detecting true differences due to lack of statistical power.

The main strengths of this study include the evaluation of a real-world population of elderly polypathological patients with acute heart failure and the incorporation of frailty assessment in addition to chronological age. Our findings may help to reduce therapeutic inertia regarding the prescription of SGLT2i in frail older adults. Moreover, this study should be regarded as hypothesis-generating, supporting the use of SGLT2i regardless of frailty status while identifying potential therapeutic targets beyond pharmacological treatment, including nutritional, functional, and social interventions, to improve prognosis and quality of life in patients with severe frailty.

## 5. Conclusions

- No significant interaction was found between SPPB score and SGLT2i use, indicating that functional frailty did not significantly modify the association between SGLT2i treatment and clinical outcomes in elderly polypathological patients with acute heart failure.
- When SPPB was analysed as a qualitative variable in an exploratory analysis, a stronger association was observed in patients with SPPB ≥ 4, with a numerically similar but not significant pattern in those with SPPB < 4. This potential benefit in severely frail patients must be interpreted as hypothesis-generating.
- Prospective studies with larger sample sizes are needed to confirm these findings, particularly in the severe frailty subgroup.

## Figures and Tables

**Table 1:** Descriptive and comparative analysis of patients with SPPB < 4 according to treatment with SGLT2i.

Variable	Total (*N* = 477)	No SGLT2i (*N* = 338)	Yes SGLT2i (*N* = 139)	*p*
**Epidemiological characteristics (*n*, %)**				
**Sex (*n*, %)**				0.531
Men (*n*, %)	175 (36.7%)	127 (37.6%)	48 (34.5%)	
Women (*n*, %)	302 (63.3%)	211 (62.4%)	91 (65.5%)	
**Age (years) (mean, SD)**	85.6 (8.2)	85.8 (7.8)	84.9 (7.2)	0.242
**BMI (kg/m^2^) (mean, SD)**	27.9 (6.4)	27.7 (6.6)	28 (6)	0.687
**Comorbidities (*n*, %)**				
**Arterial hypertension (*n*, %)**	434 (91.0%)	308 (91.1%)	126 (90.6%)	0.869
**Diabetes mellitus (*n*, %)**	223 (46.8%)	143 (42.3%)	80 (57.6%)	**0.002**
**Dyslipidaemia (*n*, %)**	303 (63.5%)	216 (63.9%)	87 (62.6%)	0.934
**Atrial fibrillation (*n*, %)**	351 (73.6%)	255 (75.4%)	96 (69.1%)	0.151
**Chronic kidney disease (*n*, %)**	237 (49.7%)	171 (50.6%)	66 (47.5%)	0.537
**Ischaemic heart disease (*n*, %)**	148 (31.0%)	103 (30.5%)	45 (32.4%)	0.683
**Chronic respiratory disease (Asthma/COPD/OSA) (*n*, %)**	164 (34.4%)	115 (34.0%)	49 (35.3%)	0.797
**Chronic liver disease (*n*, %)**	18 (3.8%)	15 (4.4%)	3 (2.2%)	0.233
**Cerebrovascular disease (*n*, %)**	89 (18.7%)	67 (19.8%)	22 (15.8%)	0.334
**Neurological disease with permanent motor impairment (*n*, %)**	51 (10.7%)	38 (11.2%)	13 (9.4%)	0.544
**Peripheral artery disease (*n*, %)**	41 (8.6%)	30 (8.9%)	11 (7.9%)	0.733
**Chronic anemia (*n*, %)**	188 (39.4%)	133 (39.3%)	55 (39.6%)	0.964
**Solid or haematological malignancy (*n*, %)**	38 (8.0%)	25 (7.4%)	13 (9.4%)	0.473
**Cognitive impairment (*n*, %)**	63 (13.2%)	53 (15.7%)	10 (7.2%)	**0.013**
**Osteoarticular disease (*n*, %)**	179 (37.5%)	121 (35.8%)	58 (41.7%)	0.224
**Clinical variables**				
**NYHA functional class**				0.205
I (*n*, %)	20 (4.2%)	11 (3.3%)	9 (6.5%)	
II (*n*, %)	239 (50.1%)	179 (53.0%)	60 (43.2%)	
III (*n*, %)	194 (40.7%)	135 (39.9%)	59 (42.4%)	
IV (*n*, %)	22 (4.6%)	12 (3.6%)	10 (7.2%)	
I–II (*n*, %)	259 (54.3%)	190 (56.2%)	69 (49.6%)	
III–IV (*n*, %)	216 (45.3%)	147 (43.5%)	69 (49.6%)	
**LVEF (%) (mean, SD)**	**51.8 (11.3)**	52.3 (11.2)	50.6 (11.6)	0.139
**Functionality**				
**Barthel (median, IQR)**	60 (40–89)	60 (40–80)	60 (40–85)	0.120
**SPPB (median, IQR)**	1 (0–2)	1 (0–2)	1 (0–2)	0.120
**MNA-SF (mean, SD)**	9.5 (2.7)	9.3 (2.7)	9.7 (2.7)	0.263
**Rockwood > 4 (*n*, %)**	309 (64.8%)	222 (65.7%)	87 (62.6%)	0.420
**Handgrip strength (kg) (median, IQR)**	10.9 (5–15)	10 (5–14)	12 (6.1–18)	**0.055**
**Analytical variables**				
**NT-proBNP (pg/mL) (median, IQR)**	6027 (3066–11,300)	5624 (2739–10,700.8)	6854 (3391–12,366)	**0.073**
**CA-125 IU/mL (median, IQR)**	49.3 (21–113)	52.3 (21–110.7)	45 (21–123)	0.807
**Creatinine (mg/dL) (median, IQR)**	1.2 (0.9–1.8)	1.2 (0.9–1.8)	1.2 (0.9–1.8)	0.875
**Cholesterol (mg/dL) (mean, SD)**	138.9 (38)	141.6 (39.8)	132.6 (32.4)	**0.014**
**Albumin (g/dL) (mean, SD)**	3.5 (0.6)	3.5 (0.6)	3.5 (0.5)	0.855
**Treatment administered**				
**ACEI (*n*, %)**	86 (18.0%)	65 (19.2%)	21 (15.1%)	0.287
**ARB (*n*, %)**	87 (18.2%)	66 (19.5%)	21 (15.1%)	0.256
**ARNI (*n*, %)**	32 (6.7%)	15 (4.4%)	17 (12.2%)	**0.002**
**Beta-blockers (*n*, %)**	281 (58.9%)	189 (55.9%)	92 (66.2%)	**0.038**
**MRA (*n*, %)**	153 (32.1%)	92 (27.2%)	61 (43.9%)	**<0.001**
**SGLT2i (*n*, %)**	139 (29.1%)			
**Furosemide maximum dose (mg/day) (median, IQR)**	80 (60–120)	80 (60–120)	100 (60–160)	**0.010**
**Protein supplements (*n*, %)**	37 (7.8%)	23 (6.8%)	14 (10.1%)	0.229
**Prognostic variables**				
**Deaths (*n*, %)**	75 (15.7%)	57 (16.9%)	18 (12.9%)	0.410
**Readmissions (*n*, %)**	190 (39.8%)	142 (42.0%)	48 (34.5%)	0.220

KEY: *N*: sample size; SGLT2i: sodium-glucose cotransporter type II inhibitors; *p*: statistical significance; *n*: number; %: percentage; SD: standard deviation; BMI: body mass index; kg/m^2^: kilograms per square metre; COPD: chronic obstructive pulmonary disease; OSA: obstructive sleep apnoea; NYHA: New York Heart Association; LVEF: left ventricular ejection fraction; IQR: interquartile range; SPPB: Short Physical Performance Battery; MNA-SF: Mini Nutritional Assessment Short Form; kg: kilograms; NT-proBNP: N-terminal pro-brain natriuretic peptide; pg/mL: picograms per millilitre; CA-125: cancer antigen 125; IU/mL: international units per millilitre; mg/dL: milligrams per decilitre; /L: per litre; g/dL: grams per decilitre; ACEI: angiotensin-converting enzyme inhibitors; ARB: angiotensin II receptor blockers; ARNI: angiotensin-neprilysin receptor inhibitors; MRA: mineralocorticoid receptor antagonists; mg/day: milligrams per day.

**Table 2:** Descriptive and comparative analysis of patients with SPPB ≥ 4 according to treatment with SGLT2i.

Variable	Total (*N* = 317)	No SGLT2i (*N* = 208)	Yes SGLT2i (*N =* 109)	*p*
**Epidemiological characteristics (*n*, %)**				
**Sex (*n*, %)**				**0.005**
Men (*n*, %)	163 (51.4%)	95 (45.7%)	68 (62.4%)	
Women (*n*, %)	154 (48.6%)	113 (54.3%)	41 (37.6%)	
**Age (years) (mean, SD)**	81.4 (9.6)	82.1 (9.6)	80.1 (9.6)	**0.078**
**BMI (kg/m^2^) (mean, SD)**	28.5 (5.8)	27.9 (5.4)	29.4 (6.4)	**0.032**
**Comorbidities (*n*, %)**				
**Arterial hypertension (*n*, %)**	284 (89.6%)	182 (87.5%)	102 (93.6%)	0.113
**Diabetes mellitus (*n*, %)**	137 (43.2%)	72 (34.6%)	65 (59.6%)	**<0.001**
**Dyslipidaemia (*n*, %)**	196 (61.8%)	125 (60.1%)	71 (65.1%)	0.380
**Atrial fibrillation (*n*, %)**	207 (65.3%)	127 (61.1%)	80 (73.4%)	**0.028**
**Chronic kidney disease (*n*, %)**	150 (47.3%)	93 (44.7%)	57 (52.3%)	0.199
**Ischaemic heart disease (*n*, %)**	100 (31.5%)	65 (31.3%)	35 (32.1%)	0.876
**Chronic respiratory disease (Asthma/COPD/OSA) (*n*, %)**	117 (36.9%)	73 (35.1%)	44 (40.4%)	0.372
**Chronic liver disease (*n*, %)**	28 (8.8%)	17 (8.2%)	11 (10.1%)	0.567
**Cerebrovascular disease (*n*, %)**	41 (12.9%)	25 (12.0%)	16 (14.7%)	0.503
**Neurological disease with permanent motor impairment (*n*, %)**	6 (1.9%)	6 (2.9%)	0 (0%)	**0.097**
**Peripheral artery disease (*n*, %)**	34 (10.7%)	19 (9.1%)	15 (13.8%)	0.206
**Chronic anemia (*n*, %)**	94 (29.7%)	61 (29.3%)	33 (30.3%)	0.861
**Solid or haematological malignancy (*n*, %)**	23 (7.3%)	21 (10.1%)	2 (1.8%)	**0.007**
**Cognitive impairment (*n*, %)**	10 (3.2%)	4 (1.9%)	6 (5.5%)	**0.098**
**Osteoarticular disease (*n*, %)**	71 (22.4%)	45 (21.6%)	26 (23.9%)	0.669
**Clinical variables**				
**NYHA functional class**				0.664
I (*n*, %)	29 (9.1%)	16 (7.7%)	13 (11.9%)	
II (*n*, %)	190 (59.9%)	126 (60.6%)	64 (58.7%)	
III (*n*, %)	90 (28.4%)	61 (29.3%)	29 (26.6%)	
IV (*n*, %)	8 (2.5%)	5 (2.4%)	3 (2.8%)	
I–II (*n*, %)	219 (69.1%)	142 (68.3%)	77 (70.6%)	
III–IV (*n*, %)	98 (30.9%)	66 (31.7%)	32 (29.4%)	
**LVEF (%) (mean, SD)**	**51 (12.5)**	52.8 (11.9)	47.8 (13.2)	**0.001**
**Functionality**				
**Barthel (median, IQR)**	95 (85–100)	95 (85–100)	100 (85–100)	0.247
**SPPB (median, IQR)**	6 (5–8)	6 (5–8)	6 (5–9)	0.191
**MNA-SF (mean, SD)**	11.2 (2.1)	11.2 (2.2)	11.2 (2)	0.836
**Rockwood > 4 (*n*, %)**	68 (21.5%)	50 (24.0%)	18 (16.5%)	0.115
**Handgrip strength (kg) (median, IQR)**	15 (10–22.5)	15 (10–21.7)	18 (11–23.6)	0.423
**Analytical variables**				
**NT-proBNP (pg/mL) (median, IQR)**	5012 (2588–9408)	4633 (2489.5–8311.5)	5849 (3198–11,086)	**0.005**
**CA-125 IU/mL (median, IQR)**	46.9 (21–90)	41 (19.5–89.7)	52.2 (23.2–101.5)	0.165
**Creatinine (mg/dL) (median, IQR)**	1.2 (0.9–1.7)	1.2 (0.9–1.6)	1.2 (0.9–1.7)	0.779
**Cholesterol (mg/dL) (mean, SD)**	137 (37.2)	137 (35.9)	136.9 (39.8)	0.985
**Albumin (g/dL) (mean, SD)**	3.5 (0.5)	3.5 (0.5)	3.6 (0.5)	0.395
**Treatment administered**				
**ACEI (*n*, %)**	75 (23.7%)	53 (25.5%)	22 (20.2%)	0.311
**ARB (*n*, %)**	77 (24.3%)	51 (24.5%)	26 (23.9%)	0.930
**ARNI (*n*, %)**	34 (10.7%)	11 (5.3%)	23 (21.1%)	**<0.001**
**Beta-blockers (*n*, %)**	208 (65.6%)	123 (59.1%)	85 (78.0%)	**0.001**
**MRA (*n*, %)**	115 (36.3%)	66 (31.7%)	49 (45.0%)	**0.015**
**SGLT2i (*n*, %)**	109 (34.4%)			
**Furosemide maximum dose (mg/day) (median, IQR)**	80 (60–120)	60 (60–100)	80 (60–120)	**<0.001**
**Protein supplements (*n*, %)**	21 (6.6%)	16 (7.7%)	5 (4.6%)	0.300
**Prognostic variables**				
**Deaths (*n*, %)**	29 (9.1%)	25 (12.0%)	4 (3.7%)	**0.018**
**Readmissions (*n*, %)**	127 (40.1%)	94 (45.2%)	33 (30.3%)	**0.021**

KEY: *N*: sample size; SGLT2i: sodium-glucose cotransporter type II inhibitors; *p*: statistical significance; *n*: number; %: percentage; SD: standard deviation; BMI: body mass index; kg/m^2^: kilograms per square metre; COPD: chronic obstructive pulmonary disease; OSA: obstructive sleep apnoea; NYHA: New York Heart Association; LVEF: left ventricular ejection fraction; IQR: interquartile range; SPPB: Short Physical Performance Battery; MNA-SF: Mini Nutritional Assessment Short Form; kg: kilograms; NT-proBNP: N-terminal pro-brain natriuretic peptide; pg/mL: picograms per millilitre; CA-125: cancer antigen 125; IU/mL: international units per millilitre; mg/dL: milligrams per decilitre; /L: per litre; g/dL: grams per decilitre; ACEI: angiotensin-converting enzyme inhibitors; ARB: angiotensin II receptor blockers; ARNI: angiotensin-neprilysin receptor inhibitors; MRA: mineralocorticoid receptor antagonists; mg/day: milligrams per day.

**Table 3:** Cox regression models of the association between SGLT2i use and the primary outcome in elderly polypathological patients with acute heart failure and SPPB test ≥ 4.

Model	HR	CI 95%	*p*
**Crude**	0.630	0.423–0.938	**0.023**
**Fully Adjusted**	0.652	0.418–1.018	**0.060**
**Adjusted without ARNI**	0.624	0.401–0.972	**0.037**

KEY: HR: hazard ratio; CI: confidence interval; *p*: statistical significance; ARNI: angiotensin receptor neprilysin inhibitor.

**Table 4:** Sensitivity analysis: propensity-score overlap weighting of the association between SGLT2i use and the primary outcome in elderly polypathological patients with acute heart failure and SPPB test ≥ 4.

Model	HR	CI 95%	*p*
**With ARNI**	0.660	0.430–1.030	**0.066**
**Without ARNI**	0.640	0.410–0.990	**0.044**

KEY: HR: hazard ratio; CI: confidence interval; *p*: statistical significance; ARNI: angiotensin receptor neprilysin inhibitor.

## Data Availability

The data presented in this study are available on request from the corresponding author. The data are not publicly available due to privacy restrictions.

## Supplementary Materials

The following supporting information can be downloaded at: https://www.mdpi.com/article/10.3390/jcm15207769/s1, Table S1. Multivariate analysis of the association between SGLT2i use and the primary outcome in elderly patients with acute heart failure and an SPPB score ≥ 4. Table S2. Multivariate Cox regression analysis excluding ARNI therapy of the association between SGLT2i use and the primary outcome in elderly patients with acute heart failure and an SPPB score ≥ 4. Table S3. Propensity Score Overlap Weighting analysis of the association between SGLT2i use and the primary outcome in elderly patients with acute heart failure and an SPPB score ≥ 4. Table S4. Propensity Score Overlap Weighting analysis (without ARNI) of the association between SGLT2i use and the primary outcome in elderly patients with acute heart failure and an SPPB score ≥ 4. Table S5. Missing data for study variables (*N* = 870, prior to exclusion for missing SPPB).

## Abbreviations

The following abbreviations are used in this manuscript:

ACEI	Angiotensin-converting enzyme inhibitors
AHF	Acute heart failure
ARB	Angiotensin II receptor blockers
ARNI	Angiotensin receptor-neprilysin inhibitor(s)
BMI	Body mass index
CA-125	Cancer antigen 125
CI	Confidence interval
COPD	Chronic obstructive pulmonary disease
g/dL	Grams per decilitre
HF	Heart failure
HR	Hazard ratio
IU/mL	international units per millilitre
IQR	Interquartile range
Kg	kilograms
kg/m^2^	kilograms per square metre
LVEF	Left ventricular ejection fraction
mg/dL	milligrams per decilitre
MNA-SF	Mini Nutritional Assessment Short Form
MRA	Mineralocorticoid receptor antagonists
*N*	Sample size
*n*	Number
NT-proBNP	N-terminal pro-B-type natriuretic peptide
NYHA	New York Heart Association
OSA	Obstructive sleep apnoea
pg/mL	Picograms per millilitre
p	Statistical significance (*p*-value)
SD	Standard deviation
SGLT2i	Sodium-glucose cotransporter type 2 inhibitors
SPPB	Short Physical Performance Battery
/L	per litre
%	percentage
