# Supplementary material for: Impact of Functional Frailty Measured by Short Physical Performance Battery Test on the Effectiveness of SGLT2 Inhibitors in Elderly Patients with Acute Heart Failure:PROFUND-IC Registry
Source: J Clin Med. 2026 Oct 9;15(20):7769. doi: 10.3390/jcm15207769 (PMC13705070; doi:10.3390/jcm15207769)
Supplement: Supplementary file 1 [file jcm-15-07769-s001.zip › jcm-4513424-supplementary.pdf]

Supplementary Materials: The following supporting information can be downloaded at https://www.mdpi.com/article/doi/s1

Table S1. Multivariate analysis of the association between SGLT2i use and the primary outcome in elderly patients with acute heart failure and an SPPB score ≥4.

Variable	HR	CI 95%	p
**SGLT2i**	0.652	0.418-1.018	0.060
**Sex**	1.199	0.812-1.771	0.362
**Age (years)**	0.980	0.961-1	0.047
**BMI (kg/m2)**	0.983	0.949-1.021	0.394
**Diabetes Mellitus**	1.165	0.788-1.722	0.444
**Atrial fibrillation**	1.123	0.752-1.675	0.571
**Cognitive impairment**	2.496	0.912-6.826	0.075
**Neurological disease with permanent motor impairment**	0.196	0.024-1.603	0.128
**Solid or haematological malignancy**	0.867	0.394-1.907	0.723
**NT-proBNP (pg/mL)**	1	1-1	0.395
**LVEF (%)**	1.001	0.982-1.020	0.922
**ARNI**	0.596	0.271-1.312	0.199
**Beta-blockers**	0.857	0.575-1.277	0.449
**MRA**	0.824	0.551-1.234	0.348
**Furosemide maximum dose (mg/day)**	1.001	0.998-1.003	0.566

KEY: HR: hazard ratio; CI: confidence interval; p: statistical significance; SGLT2i : sodium-glucose cotransporter type II inhibitors; BMI: body mass index; kg/m²: kilograms per square metre; NT-proBNP: N-terminal pro-B-type natriuretic peptide; pg/mL: picograms per millilitre; LVEF: left ventricular ejection fraction; ARNI: angiotensin receptor neprilysin inhibitor; mg/day: milligrams per day.
NOTE: The hazard ratio for NT-proBNP is reported per 1 pg/mL increase; given the wide range of this biomarker in the cohort, the estimate rounds to 1.000 despite reflecting a real, non-zero association. NT-proBNP was entered as a continuous, untransformed variable.

Table S2. Multivariate Cox regression analysis excluding ARNI therapy of the association between SGLT2i use and the primary outcome in elderly patients with acute heart failure and an SPPB score ≥4.

Variable	HR	CI 95%	p
**SGLT2i**	0.624	0.401-0.972	**0.037**
**Sex**	1.216	0.824-1.795	0.325
**Age (years)**	0.979	0.960-0.998	0.030
**BMI (kg/m2)**	0.984	0.949-1.021	0.394
**Diabetes Mellitus**	1.149	0.778-1.698	0.486
**Atrial fibrillation**	1.122	0.752-1.675	0.574

**Cognitive impairment**	2.614	0.959-7.125	0.060
**Neurological disease with permanent motor impairment**	0.190	0.023-1.560	0.122
**Solid or haematological malignancy**	0.854	0.387-1.882	0.695
**NT-proBNP (pg/mL)**	1	1-1	0.322
**LVEF (%)**	1.005	0.987-1.023	0.592
**Beta-blockers**	0.839	0.563-1.250	0.388
**MRA**	0.804	0.538-1.204	0.290
**Furosemide maximum dose (mg/day)**	1.001	0.998-1.003	0.654

KEY: HR: hazard ratio; CI: confidence interval; p: statistical significance; SGLT2i : sodium-glucose cotransporter type II inhibitors; BMI: body mass index; kg/m^2: kilograms per square metre; NT-proBNP: N-terminal pro-B-type natriuretic peptide; pg/mL: picograms per millilitre; LVEF: left ventricular ejection fraction; ARNI: angiotensin receptor neprilysin inhibitor; mg/day: milligrams per day.

NOTE: The hazard ratio for NT-proBNP is reported per 1 pg/mL increase; given the wide range of this biomarker in the cohort, the estimate rounds to 1.000 despite reflecting a real, non-zero association. NT-proBNP was entered as a continuous, untransformed variable.

Table S3. Propensity Score Overlap Weighting analysis of the association between SGLT2i use and the primary outcome in elderly patients with acute heart failure and an SPPB score ≥4.

Variable	Overall (*N* = 103)	No SGLT2i (*N* = 52)	Yes SGLT2i (*N* = 52)
**Sex (*n*, %)**			
Men (*n*, %)	60 (58%)	30 (58%)	30 (58%)
Women (*n*, %)	45 (42%)	23 (42%)	23 (42%)
**Age (years) (mean, SD)**	81 (10)	81 (10)	81 (10)
**BMI (kg/m2) (mean, SD)**	28.8 (5.5)	28.8 (5.5)	28.8 (5.6)
**Diabetes Mellitus (*n*, %)**	50 (48%)	25 (48%)	25 (48%)
**Atrial fibrillation (*n*, %)**	73 (71%)	37 (71%)	37 (71%)
**Cognitive impairment (*n*, %)**	3 (2.6%)	1 (2.6%)	1 (2.6%)
**Neurological disease with permanent motor impairment (*n*, %)**	0 (<0.1%)	0 (<0.1%)	0 (0%)
**Solid or haematological malignancy (*n*, %)**	3 (2.8%)	1 (2.8%)	1 (2.8%)

**NT-proBNP (pg/mL) (mean, SD)**	7544 (6905)	7544 (7649)	7544 (6098)
**LVEF (%) (mean, SD)**	50 (13)	50 (13)	50 (13)
**ARNI (*n*, %)**	14 (13%)	7 (13%)	7 (13%)
**Beta-blockers (*n*, %)**	78 (73%)	38 (73%)	38 (73%)
**MRA (*n*, %)**	43 (42%)	21 (42%)	22 (41.6%)
**Furosemide maximum dose (mg/day) (mean, SD)**	109 (81)	109 (87)	109 (75)

KEY: SGLT2i: sodium-glucose cotransporter type II inhibitors; BMI: body mass index; kg/m^2: kilograms per square metre; NT-proBNP: N-terminal pro-B-type natriuretic peptide; pg/mL: picograms per millilitre; LVEF: left ventricular ejection fraction; ARNI: angiotensin receptor neprilysin inhibitor; mg/day: milligrams per day.

Table S4. Propensity Score Overlap Weighting analysis (without ARNI) of the association between SGLT2i use and the primary outcome in elderly patients with acute heart failure and an SPPB score ≥4.

Variable	Overall (*N* = 105)	No SGLT2i (*N* = 53)	Yes SGLT2i (*N* = 53)
**Sex (*n*, %)**			
Men (*n*, %)	60 (57%)	30 (57%)	30 (57%)
Women (*n*, %)	45 (43%)	23 (43%)	23 (43%)
**Age (years) (mean, SD)**	81 (10)	81 (10)	81 (9)
**BMI (kg/m2) (mean, SD)**	28.8 (5.5)	28.8 (5.5)	28.8 (5.6)
**Diabetes Mellitus (*n*, %)**	51 (48%)	25 (48%)	25 (48%)
**Atrial fibrillation (*n*, %)**	75 (71%)	38 (71%)	38 (71%)
**Cognitive impairment (*n*, %)**	3 (2.6%)	1 (2.6%)	1 (2.6%)
**Neurological disease with permanent motor impairment (*n*, %)**	0 (<0.1%)	0 (<0.1%)	0 (0%)
**Solid or haematological malignancy (*n*, %)**	3 (2.6%)	1 (2.6%)	1 (2.6%)
**NT-proBNP (pg/mL) (mean, SD)**	7543 (6876)	7543 (7674)	7543 (6000)
**LVEF (%) (mean, SD)**	49 (13)	49 (13)	49 (13)
**Beta-blockers (*n*, %)**	78 (74%)	39 (74%)	39 (74%)
**MRA (*n*, %)**	44 (41%)	21 (41%)	21 (41.3%)
**Furosemide maximum dose (mg/day) (mean, SD)**	108 (80)	108 (85)	108 (74)

KEY: SGLT2i: sodium-glucose cotransporter type II inhibitors; BMI: body mass index; kg/m^2: kilograms per square metre; NT-proBNP: N-terminal pro-B-type natriuretic peptide; pg/mL: picograms per millilitre; LVEF: left ventricular ejection fraction; ARNI: angiotensin receptor neprilysin inhibitor; mg/day: milligrams per day.

Table S5. Missing data for study variables (*N* = 870, prior to exclusion for missing SPPB).

Variable	N missing	% missing
**SPPB score**	76	8.7%
**SGLT2i use**	0	0%
**Age**	0	0%
**Sex**	0	0%
**Diabetes mellitus**	0	0%
**Creatinine**	0	0%
**Beta-blockers use**	0	0%
**Cognitive impairment**	0	0%
**ACEI use**	1	0.1%
**Arterial Hypertension**	1	0.1%
**Chronic kidney disease**	1	0.1%
**Atrial fibrillation**	1	0.1%
**NT-proBNP**	2	0.2%
**ARB use**	2	0.2%
**Dyslipidaemia**	2	0.2%
**ARNI use**	2	0.2%
**NYHA functional class**	3	0.3%
**Protein supplements**	4	0.5%
**MRA use**	5	0.6%
**Barthel Index**	6	0.7%
**LVEF**	9	1.0%
**Rockwood frailty index**	17	2.0%
**Maximum daily furosemide dose**	28	3.2%
**BMI**	36	4.1%
**Cholesterol**	57	6.6%
**Albumin**	197	22.6%
**CA-125**	234	26.9%
**MNA-SF**	388	44.6%
**Handgrip strength**	475	54.6%

KEY: SPPB: Short Physical Performance Battery; SGLT2i: sodium-glucose cotransporter type 2 inhibitors; ACEI: angiotensin-converting enzyme inhibitors; NT-proBNP: N-terminal pro-brain natriuretic peptide; ARB: angiotensin II receptor blockers; ARNI: angiotensin-neprilysin receptor inhibitors; NYHA: New York Heart Association; MRA: mineralocorticoid receptor antagonists; LVEF: left ventricular

ejection fraction; BMI: body mass index; CA-125: cancer antigen 125; MNA-SF: Mini Nutritional Assessment Short Form.
